# Supplementary material for: Complementarity in Allen’s and Bergmann’s rules among birds
Source: Nat Commun. 2023 Jul 15;14:4240. doi: 10.1038/s41467-023-39954-9 (PMC10349823; doi:10.1038/s41467-023-39954-9)
Supplement: Supplementary file 1 — Supplementary Information [file 41467_2023_39954_MOESM1_ESM.pdf]

Supplementary Information

Title: Complementarity in Allen's and Bergmann's rules among birds

Authors: Justin W. Baldwin[1*], Joan Garcia-Porta[1,2,3*], Carlos A. Botero[1,4]

Affiliations and present addresses:

[1] Department of Biology, Washington University, St. Louis, MO 63130, USA.

[2] Departament de Genètica Microbiologia i Estadística, Facultat de Biologia & Institut de Recerca de la Biodiversitat (IRBio), Universitat de Barcelona, Spain

[3] Department of Biodiversity, Ecology and Evolution, Complutense University of Madrid, Madrid, Spain

[4] Department of Integrative Biology, University of Texas at Austin, Austin, TX 78712

*These authors contributed equally to this work

Corresponding author: Justin Baldwin: jwbaldwin@wustl.edu

Code availability. Code is available on Zenodo under doi: 10.5281/zenodo.8092265

Table of Contents

Supplementary Table 1 ……………………………………………………………………p. 3
Supplementary Table 2 ……………………………………………………………………p. 4
Supplementary Table 3 ……………………………………………………………………p. 5
Supplementary Table 4 ……………………………………………………………………p. 6
Supplementary Table 5 ……………………………………………………………………p. 7
Supplementary Figure 1 ……………………………………………………………………p. 8
Supplementary Figure 2 ……………………………………………………………………p. 9
Supplementary Figure 3 ……………………………………………………………………p. 10
Supplementary Figure 4 ……………………………………………………………………p. 11
Supplementary Figure 5 ……………………………………………………………………p. 12-13
Supplementary Figure 6 ……………………………………………………………………p. 14-15
Supplementary Figure 7 ……………………………………………………………………p. 16
Supplementary Figure 8 ……………………………………………………………………p. 17
Supplementary Figure 9 ……………………………………………………………………p. 18-19
Supplementary Figure 10 ……………………………………………………………………p. 20
Supplementary References ……………………………………………………………………p. 21

Supplementary Table 1 | Bill size scales nonlinearly with body size. Coefficient tables from linear and non-linear models of the bill-body allometry shown in Fig. 3a. Models are species-level phylogenetic regressions (N = 6,974) with Pagel's branch-length transformations and species-trees trees with the Hackett backbone[1,2]. Two-sided effects tests on model intercepts and slopes are reported with effect sizes, standard errors, t-statistics and p-values.

Model of bill-body allometry	Term	Effect Size	Standard Error	T-statistic	P value
Linear	Intercept	0.644	0.063	10.274	<0.0001
	Slope of log body mass	0.160	0.002	81.149	<0.0001
	AIC	-17721			
	Adjusted R-squared	0.486			
	Lambda	0.960			
Nonlinear	Intercept	0.352	0.057	6.171	<0.0001
	Slope of log body mass	0.329	0.006	58.657	<0.0001
	Slope of $(\log \text{body mass})^2$	-0.020	0.001	-31.812	<0.0001
	AIC	-18654			
	Adjusted R-squared	0.5559			
	Lambda	0.949			

Supplementary Table 2 | Observed rule conformance across different methodological variants in our analyses. As in the main text, conformance is defined as 95% credible intervals of the posterior random slope that do not overlap zero and exhibit the trend predicted by either Allen (positive) or Bergmann (negative). Cell counts denote numbers of families that conform to each rule. Cochran-Mantel-Haenszel tests[3] evaluate potential differences in cell counts between the counts of families in the different conformance categories between models that used specific methodological choices and the baseline methodology used in the main text (first row). Labels for methodological variants indicate differences with the baseline methodology. X^2 denotes Mantel-Haenszel Chi-squared value with 1 degree of freedom for each test and associated two-sided p-values are shown.

	Rule conformance					
**Methodological variant**	**Neither rule**	**Allen's rule**	**Bergmann's rule**	**Both rules**	**CMH test**	
					**X^2**	**P-value**
Non-phylogenetic PCA / absolute bill size (linear)	90 (36)	9	8	0	Baseline	
Relative bill size	80 (40)	19	6	2	0.03	0.854
Phylogenetic PCA	90 (35)	9	8	0	1.57	0.210
Alternative bill size metric (centroid size)	58 (32)	4	4	0	1.06	0.304
Thermal ranges > 10 °C	64 (24)	9	8	0	1.88	0.170
Minimum 20 species per family	65 (24)	9	8	0	1.87	0.172
Orders	14 (3)	2	5	0	1.43	0.231
Phylogenetic hypothesis with Ericson backbone	89 (34)	9	8	0	1.68	0.195

Supplementary Table 3 | Family-level conformance to Allen's and Bergmann's rules at different taxonomic levels. Numbers indicate the number of families in each category when absolute bill size (i.e., non-phylogenetic PC1 derived from linear metrics) and a summary tree with the Hackett backbone[1,2] were used in our analyses.

	**Rule conformance**			
**Clade**	**Allen**	**Bergmann**	**Both**	**None**
Class Aves	9	8	0	90
Passeriformes	**7**	1	0	**60**
Non-passerines	2	7	0	30

Supplementary Table 4 | Variance components of principal component analysis on 3D geometric morphometrics of 3,512 species. Principal components (PC) one through eight are shown.

	**PC1**	**PC2**	**PC3**	**PC4**	**PC5**	**PC6**	**PC7**	**PC8**
Eigenvalues	0.0254	0.0133	0.0025	0.001	0.0007	0.0006	0.0004	0.0002
Proportion of variance	0.5690	0.2969	0.0570	0.0231	0.0146	0.0129	0.0085	0.0042
Cumulative proportion of variance	0.5690	0.8659	0.9229	0.9460	0.9606	0.9735	0.9820	0.9862

Supplementary Table 5 | Multivariable phylogenetic regressions of strengths of Allen's and Bergmann's rules against family traits. Phylogenetic regressions were performed with family-level trees using the Hackett backbone[1,2]. Two-sided effects tests on predictors variables are reported with effect sizes, standard errors, t-statistics and p-values.

Response	Predictor	Effect Size	Standard Error	T-statistic	P value
Absolute bill slope	Mean absolute bill size	0.0013	0.0008	1.732	0.086
	Mean relative bill size	0.0002	0.0007	0.259	0.797
	Temperature range	-0.0013	0.0006	-2.318	0.022*
	ln N species per family	0.0006	0.0005	1.132	0.260
	Mean kernel density	0.0007	0.0006	1.274	0.206
Body mass slope	Mean body mass	0.0206	0.0034	6.135	0.0008*
	Mean relative bill size	0.0021	0.0027	0.781	0.437
	Temperature range	-0.0011	0.0022	-0.488	0.627
	ln N species per family	0.001	0.0022	0.452	0.652
	Mean kernel density	0.0002	0.0025	0.010	0.921

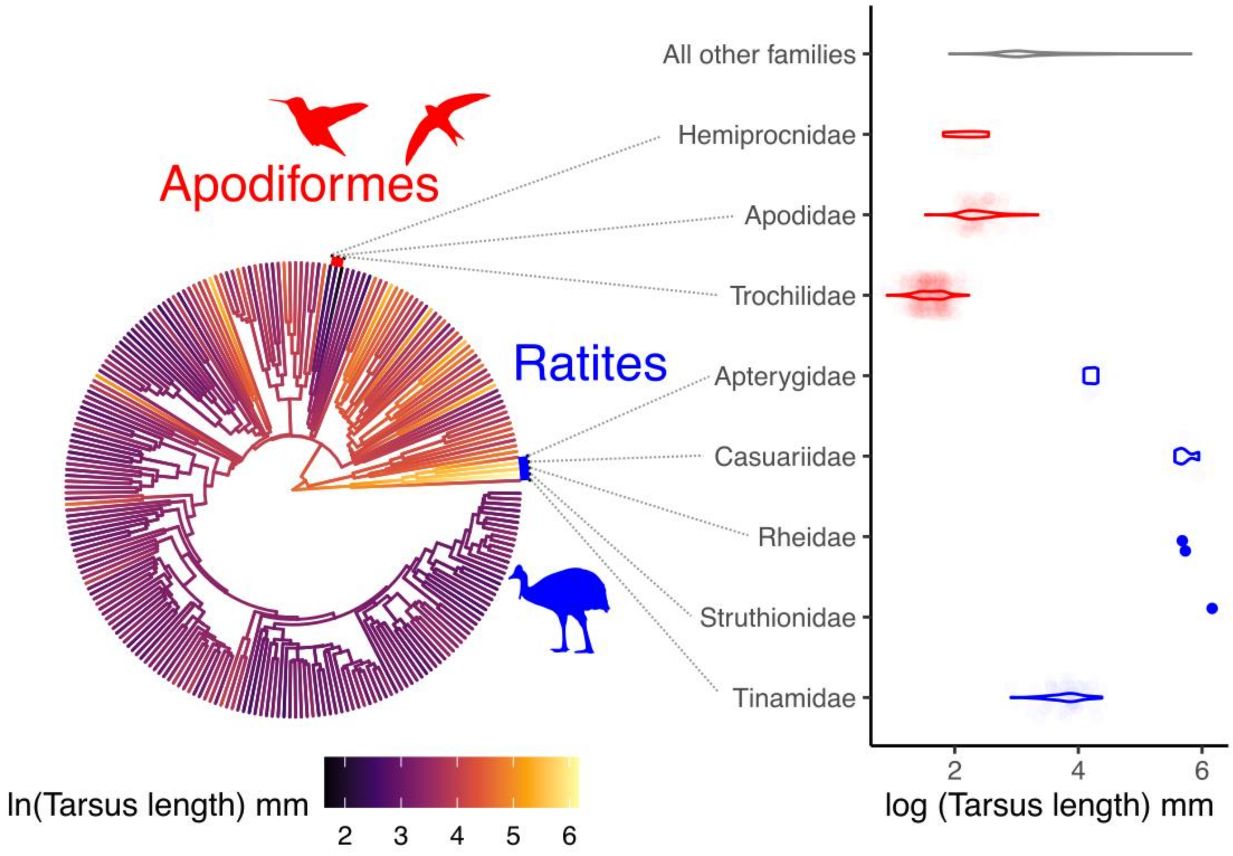

Supplementary Figure 1 | Tarsus length is variable in some bird families and highly constrained in others. Morphological data[4] indicate that tarsus length is small and constrained in the highly aerial Apodiformes (red symbols: swifts, treeswifts and hummingbirds). In contrast, tarsus length in the terrestrial ratites (blue symbols: tinamous, ostrich, rhea, cassowary, kiwi) is generally large and quite variable. All other families (black) have variable tarsus lengths. Densities are shown for groups with more than two species in our sample. We thank Ferran Sayol and Margot Michaud for submitting their artwork to Phylopic under the CC0 license.

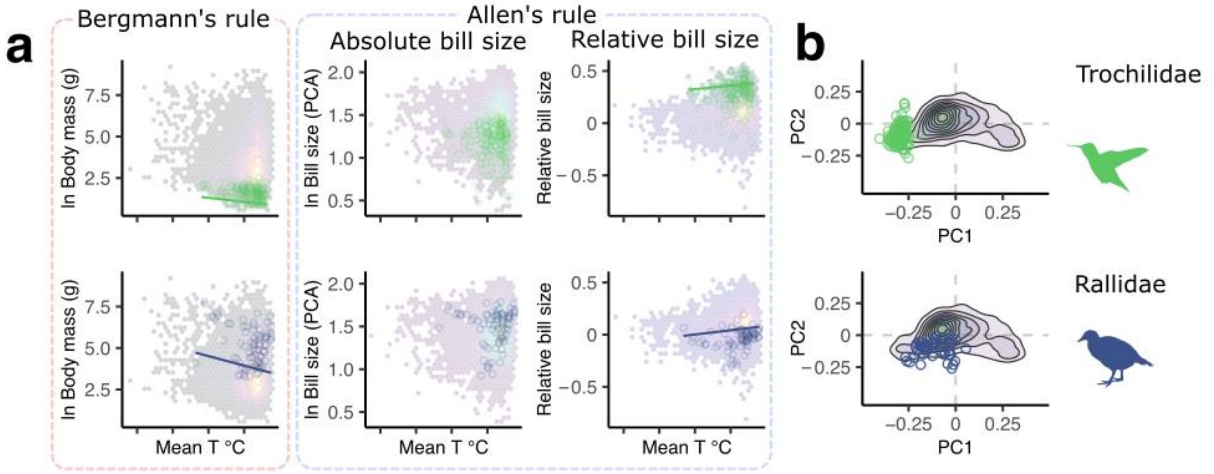

Supplementary Figure 2 | Non-existent conformity to Allen's rule is sometimes suggested by the use of relative bill size. (**a**) Some families conform to Bergmann's rule but show no changes in their absolute bill size over thermal gradients (Trochilidae, Rallidae). In these cases, analyses of relative (but not absolute) bill size can suggest that they conform to Allen's rule. (**b**) The two families in our sample that exhibit this pattern have generally rare bill shapes (i.e., their mean bill shape lies on the periphery of the morphospace), indicating that they may be ecological constrained from adapting to thermal gradients via changes in their bill (cf. Fig. 4, Table 2). We thank Ferran Sayol and Margot Michaud for submitting their artwork to Phylopic under the CC0 license.

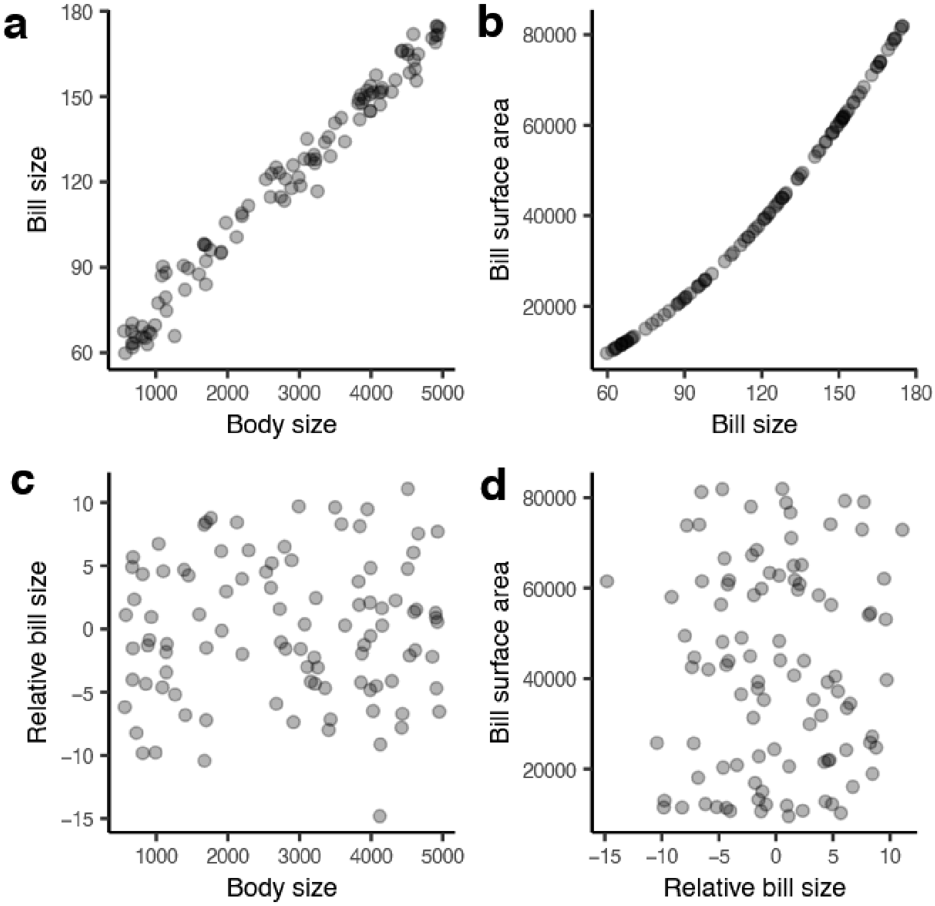

Supplementary Figure 3 | Absolute bill size is often a better proxy of surface area than relative bill size. Simulation of bill-body allometry following: Bill size = 50 + 0.025× Body size + ε (where $\varepsilon \sim N(0, 5)$) (**a**). Assuming a conical shape, surface area in (**b**) was computed as r*$\sqrt{(r^2 + bill\ length^2)} \times \pi$, where *r* is the radius at the base of the bill, defined as r = 0.7 × Bill length. Relative bill sizes (**c**) capture how absolute bill sizes differ from expectations for a given body size (i.e., the residuals from a linear model in **a**). Absolute bill size (measured here as bill length) is highly correlated with bill surface area (linear model for **b**, slope test of significance, t = 77.11, $p < 0.001$, $R^2 = 0.984$). In contrast, relative bill size is uncorrelated with either body size (**c** linear model, slope test of significance, t = 0, p = 1.0, $R^2 < 0.001$) or surface area (**d,** linear model, slope test of significance, t = 1.198, p = 0.234, $R^2 = 0.001$).

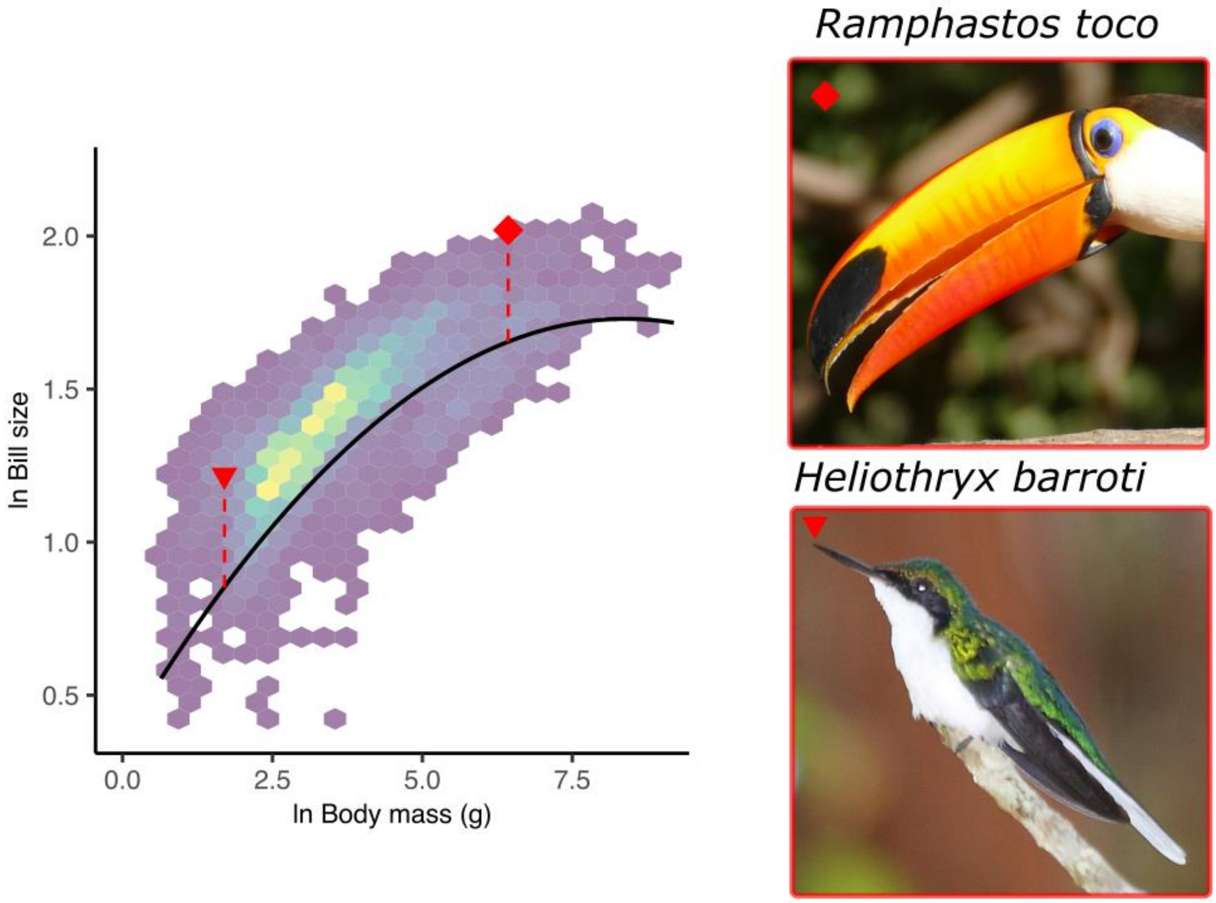

Supplementary Figure 4 | Relative bill size does not capture the surface area used for heat loss. Relative bill size is computed as the residuals from a phylogenetic regression of bill size on body size (population intercept and slope shown in orange line). Toco Toucans, *Ramphastos toco* (red rhombus) and Purple-crowned Fairy, *Heliothryx barroti*, (red triangle) have almost identical relative bill sizes (0.36) but are known to differ dramatically in both their bill's surface and their ability to dissipate heat through the bill[5,6]. We thank Dominic Sherony and Bernard Dupont for making their photos available on Wikimedia Commons under Creative Commons license CC-BY-SA – see Supplemental Information[7]; https://creativecommons.org/licenses/by/4.0/).

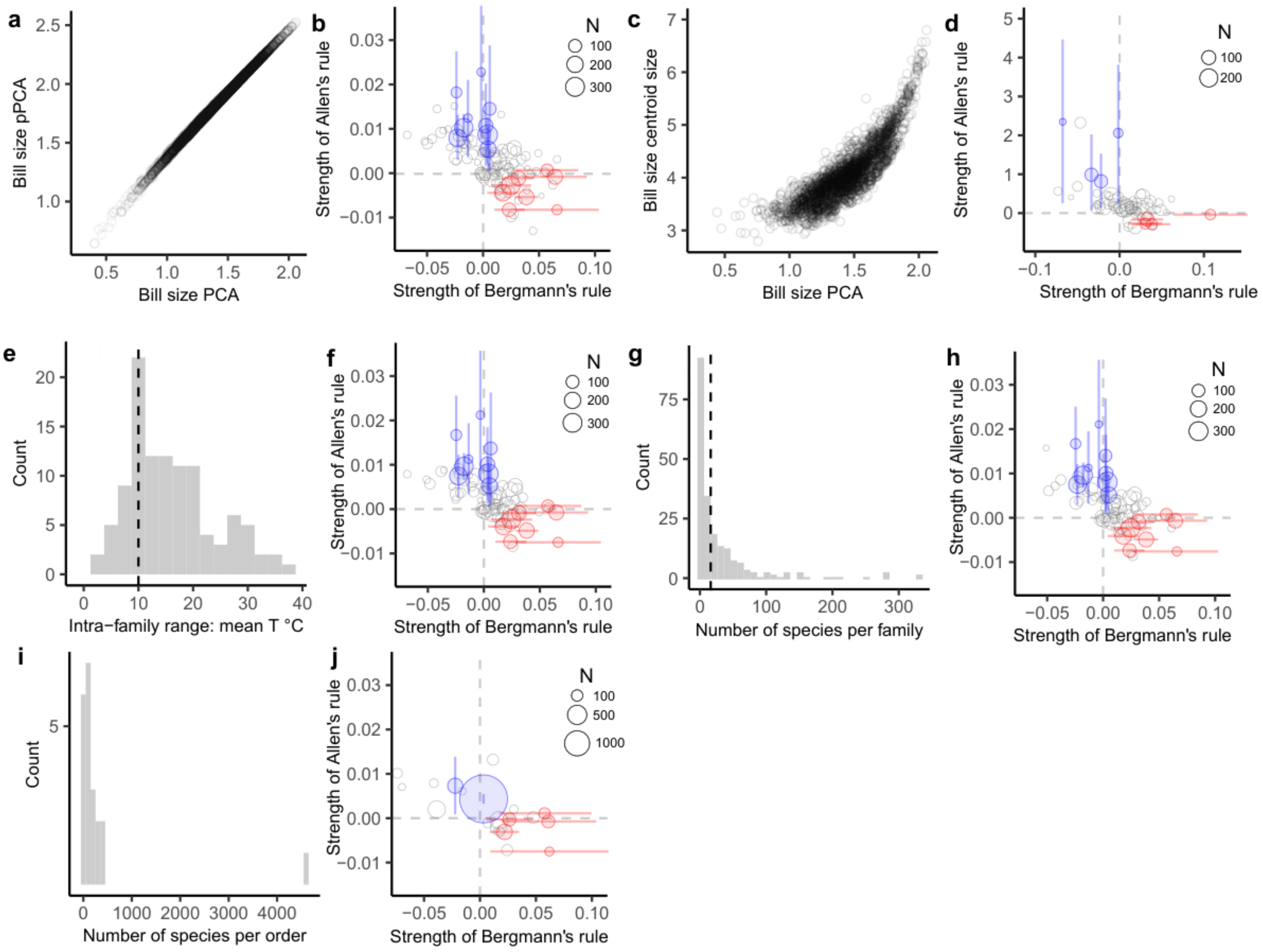

Supplementary Figure 5 | Graphic summary of alternative analyses exploring the robustness of our findings to important methodological choices. PC1 scores derived from a phylogenetic PCA are highly correlated with those obtained through non-phylogenetic PCA (**a**), and lead to qualitatively identical results as the ones presented in Fig. 3. Specifically, body size gradients continue to be inversely correlated with bill size gradients, and a clear majority of families in our sample continue to exhibit clear trends of expected surface-to-volume adjustments across thermal gradients without significant changes in either trait (**b**). As in the main text, significant conformance to Allen's and Bergmann's rules are respectively depicted in blue and red, circle size indicates the number of sampled species in a family and lines depict

95% posterior credible intervals for our slope estimates. Alternative estimates of bill size derived from the landmarks of an earlier geometric morphometric study are also highly correlated with the linear estimates used in the main text (**c**) and also yield similar findings as those presented in the main text (**d**). Similarly, excluding families that occupy narrow temperature ranges (i.e., < 10 ° C, dashed line in (**e**) does not affect our conclusions (**f**), and neither does retaining only families with 20 or more species (dashed line in **g**, resulting analysis in **h**). Likewise, rerunning the analysis at the level of biological orders yielded highly imbalanced sampling across orders (**i**), but similar results (**j**).

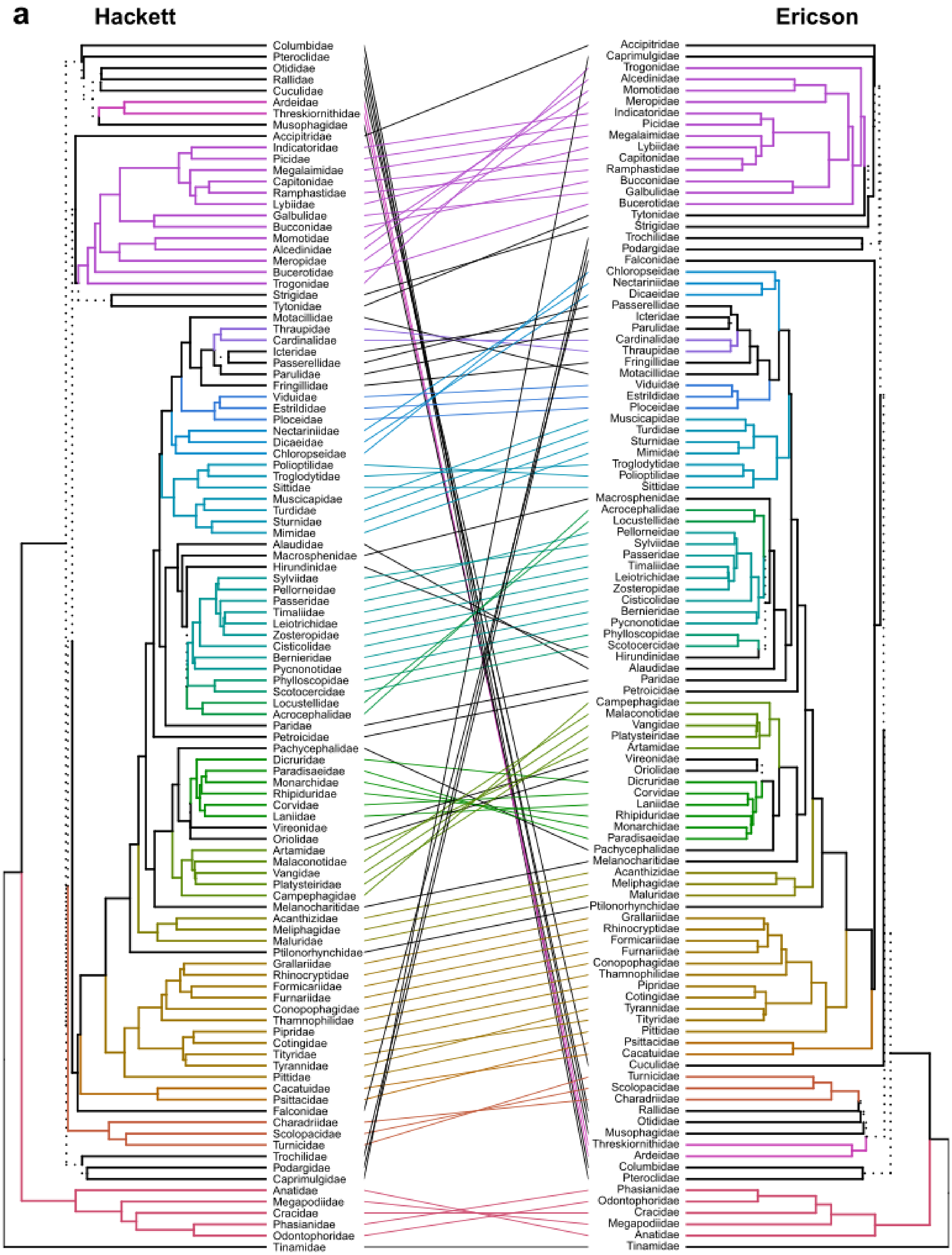
a
Hackett
Ericson
Columbidae
Pteroclidae
Otididae
Rallidae
Cuculidae
Ardeidae
Threskiornithidae
Musophagidae
Accipitridae
Indicatoridae
Picidae
Megalaimidae
Capitonidae
Ramphastidae
Lybiidae
Galbulidae
Bucconidae
Momotidae
Alcedinidae
Meropidae
Bucerotidae
Trogonidae
Strigidae
Tytonidae
Motacillidae
Thraupidae
Cardinalidae
Icteridae
Passerellidae
Parulidae
Fringillidae
Viduidae
Estrildidae
Ploceidae
Nectariniidae
Dicaeidae
Chloropseidae
Polioptilidae
Troglodytidae
Sittidae
Muscicapidae
Turdidae
Sturnidae
Mimidae
Alaudidae
Macrosphenidae
Hirundinidae
Sylviidae
Pellorneidae
Passeridae
Timaliidae
Leiotrichidae
Zosteropidae
Cisticolidae
Bernieridae
Pycnonotidae
Phylloscopidae
Scotocercidae
Locustellidae
Acrocephalidae
Paridae
Petroicidae
Pachycephalidae
Dicruridae
Paradisaeidae
Monarchidae
Rhipiduridae
Corvidae
Laniidae
Vireonidae
Oriolidae
Artamidae
Malaconotidae
Vangidae
Platysteiridae
Campephagidae
Melanocharitidae
Acanthizidae
Meliphagidae
Maluridae
Ptilonorhynchidae
Grallariidae
Rhinocryptidae
Formicariidae
Furnariidae
Conopophagidae
Thamnophilidae
Pipridae
Cotingidae
Tityridae
Tyrannidae
Pittidae
Cacatuidae
Psittacidae
Falconidae
Charadriidae
Scolopacidae
Turnicidae
Trochilidae
Podargidae
Caprimulgidae
Anatidae
Megapodiidae
Cracidae
Phasianidae
Odontophoridae
Tinamidae
Accipitridae
Caprimulgidae
Trogonidae
Alcedinidae
Momotidae
Meropidae
Indicatoridae
Picidae
Megalaimidae
Lybiidae
Capitonidae
Ramphastidae
Bucconidae
Galbulidae
Bucerotidae
Tytonidae
Strigidae
Trochilidae
Podargidae
Falconidae
Chloropseidae
Nectariniidae
Dicaeidae
Passerellidae
Icteridae
Parulidae
Cardinalidae
Thraupidae
Fringillidae
Motacillidae
Viduidae
Estrildidae
Ploceidae
Muscicapidae
Turdidae
Sturnidae
Mimidae
Troglodytidae
Polioptilidae
Sittidae
Macrosphenidae
Acrocephalidae
Locustellidae
Pellorneidae
Sylviidae
Passeridae
Timaliidae
Leiotrichidae
Zosteropidae
Cisticolidae
Bernieridae
Pycnonotidae
Phylloscopidae
Scotocercidae
Hirundinidae
Alaudidae
Paridae
Petroicidae
Campephagidae
Malaconotidae
Vangidae
Platysteiridae
Artamidae
Vireonidae
Oriolidae
Dicruridae
Corvidae
Laniidae
Rhipiduridae
Monarchidae
Paradisaeidae
Pachycephalidae
Melanocharitidae
Acanthizidae
Meliphagidae
Maluridae
Ptilonorhynchidae
Grallariidae
Rhinocryptidae
Formicariidae
Furnariidae
Conopophagidae
Thamnophilidae
Pipridae
Cotingidae
Tyrannidae
Tityridae
Pittidae
Psittacidae
Cacatuidae
Cuculidae
Turnicidae
Scolopacidae
Charadriidae
Rallidae
Otididae
Musophagidae
Threskiornithidae
Ardeidae
Columbidae
Pteroclidae
Phasianidae
Odontophoridae
Cracidae
Megapodiidae
Anatidae
Tinamidae

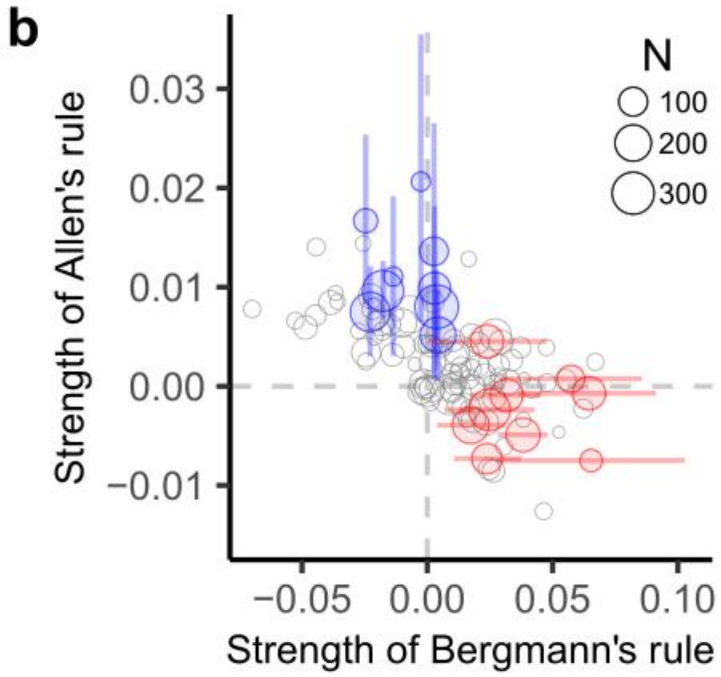

Supplementary Figure 6 | Comparison of family-level summary trees. Tanglegram (**a**) comparing the family-level summary trees derived from the Hackett[2] (left) and Ericson[7] (right) backbones. Edges link families and colours denote shared clades. Families that occupy different topological positions across trees are linked with black lines and are highlighted with dashed edges. Using summary trees with the Ericson[8] rather than the Hackett[2] backbone did not affect the results (**b**). As in the main text, significant conformance to Allen's and Bergmann's rules are respectively depicted in blue and red, circle size indicates the number of sampled species in a family and lines depict 95% posterior credible intervals for our slope estimates.

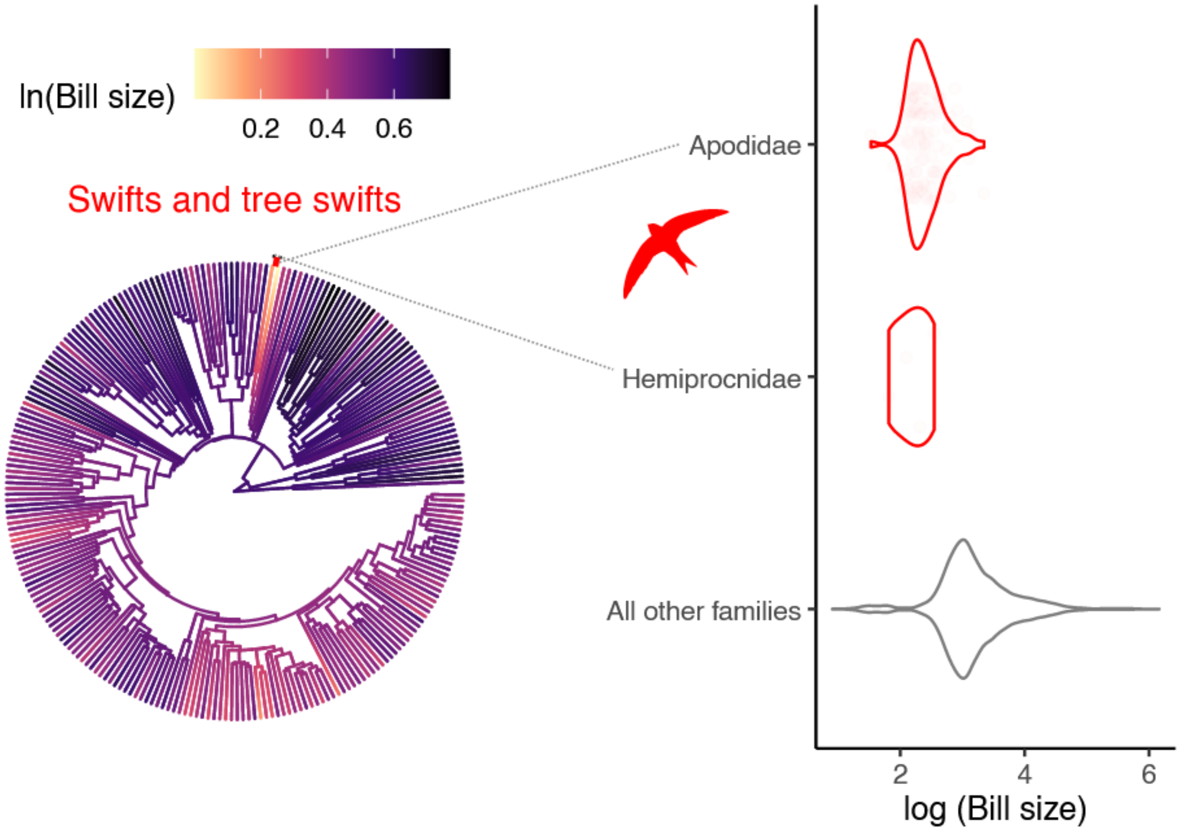

Supplementary Figure 7 | Swifts (Apodidae) and treeswifts (Hemiprocnidae) have extremely reduced bill sizes. Bill size is defined here as the first axis in a PCA of four linear bill measurements[4]. Treeswifts were excluded from our main text analyses because they include fewer than 10 sampled species, and swifts were initially excluded due to their highly atypical morphology. We thank Ferran Sayol for submitting artwork to Phylopic under a CC0 license.

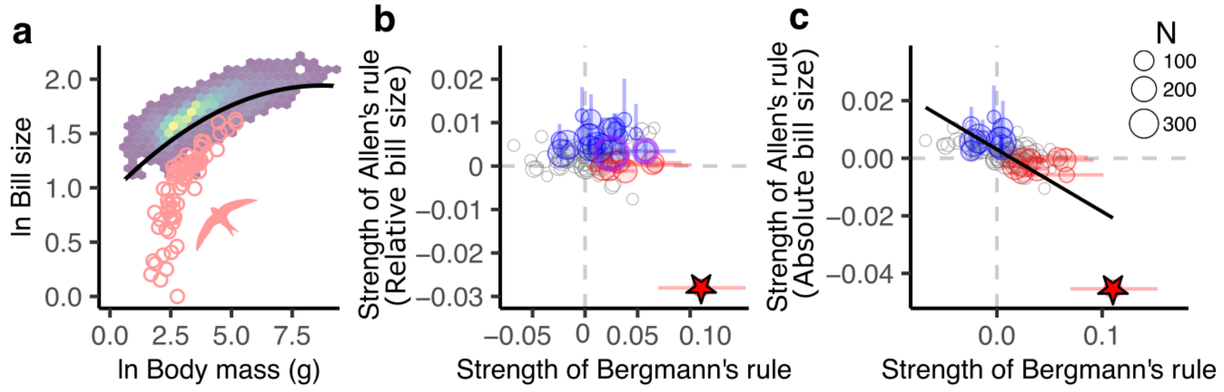

Supplementary Figure 8 | Including swifts (Apodidae) in our analyses does not alter our main findings. (a) Swifts (red circles) have atypically small exposed culmens and therefore exhibit stronger allometric scaling of the bill than most other bird families. In accordance with our main findings, this extreme bill specialization is linked in this group to a particularly strong gradient of change in body size over thermal gradients (Apodidae depicted as a star in **b** and **c**). When replicating our models including swifts in the dataset, the patterns depicted in Fig. 3 continue to be recovered for both relative bill size (**b**) and absolute bill size (**c**). Significant conformance to Allen's and Bergmann's rules in **b** and **c** are respectively depicted in blue and red, circle size indicates the number of sampled species in a family and lines depict 95% posterior credible intervals for our slope estimates. We thank Ferran Sayol for submitting artwork to Phylopic under a CC0 license.

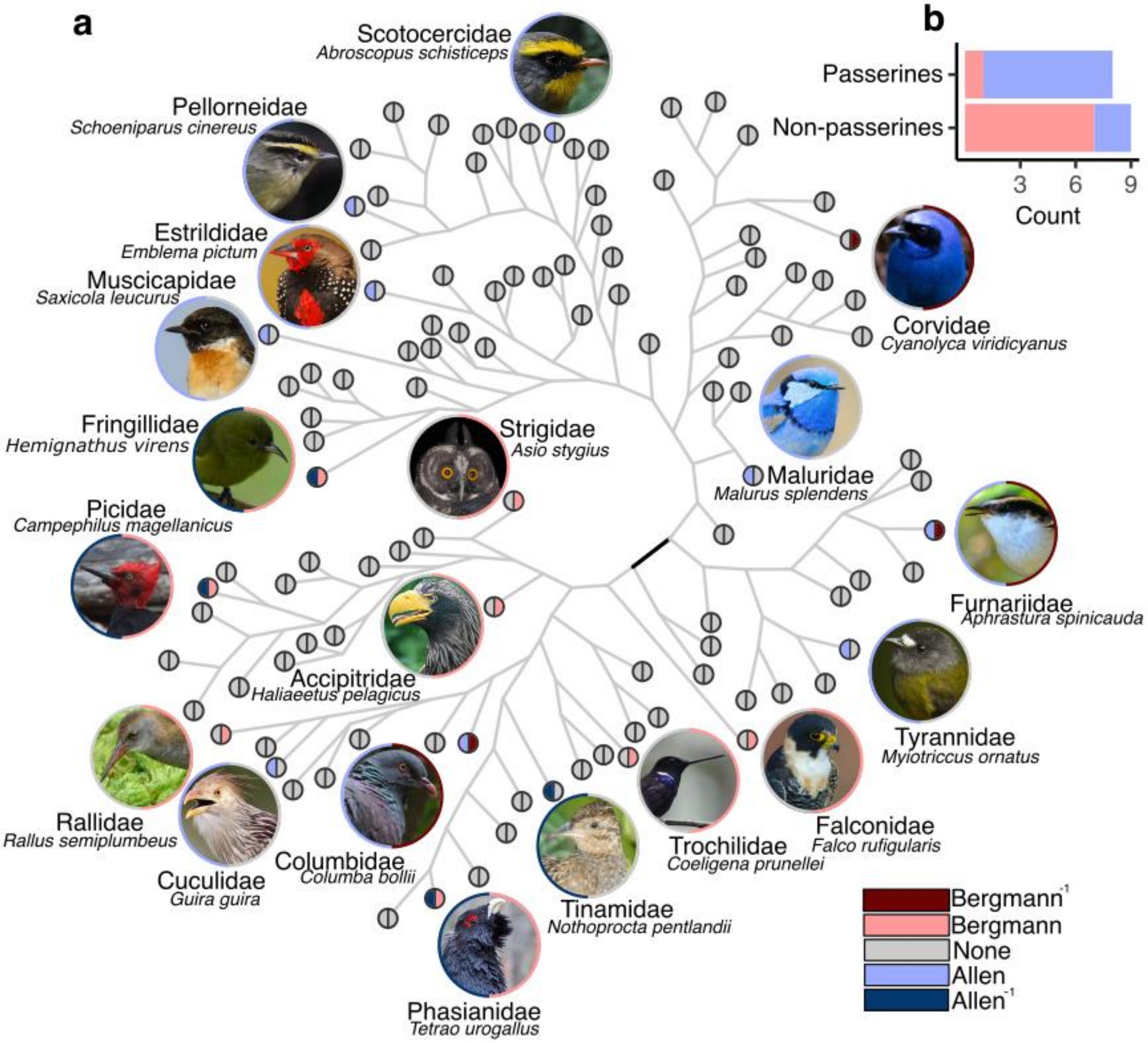

Supplementary Figure 9 | Phylogenetic distribution of surface-to-volume adjustments over thermal gradients in a variety of avian families. (**a**) Semicircles at the tips of this cladogram and around representative species depict the observed family-wide changes in bill (left semicircle) and body size (right semicircle) across geographic temperature gradients. Darker colours denote families in which significant changes in morphology are observed but in the opposite direction from Bergmann's or Allen's expectations (dubbed "$^{-1}$" in the lower right

colour legend). The black edge in the cladogram denotes the branch separating passerines (upper section of the cladogram) from non-passerines (lower section). Tree topology is an unrooted family-level tree of the Global Bird Phylogeny[1] with the Hackett backbone[2] (**b**) Relative abundance of each conformance category in passerines and non-passerines. We thank JJ Harrison, James Brennan, Hector Bottai, Carlo Benitez Gomez, Umeshsrinivasan, Félix Uribe, Imran Shah, Liam Quinn, Matěj Baťha, Neil Orlando Diaz Martinez, Dfaulder, Hobbyfotowiki, SighmanB, Dick Daniels, Joao Quental, Francesco Veronesi, Gabriel Barrera Maffioletti, Dibyendu Ash, PotMart186 and thibaudaronson for making their photos available on Wikimedia Commons under Creative Commons licenses (CC-BY-SA, CC-BY, CC0 – see Supplemental Information[7]; https://creativecommons.org/licenses/by-sa/4.0/).

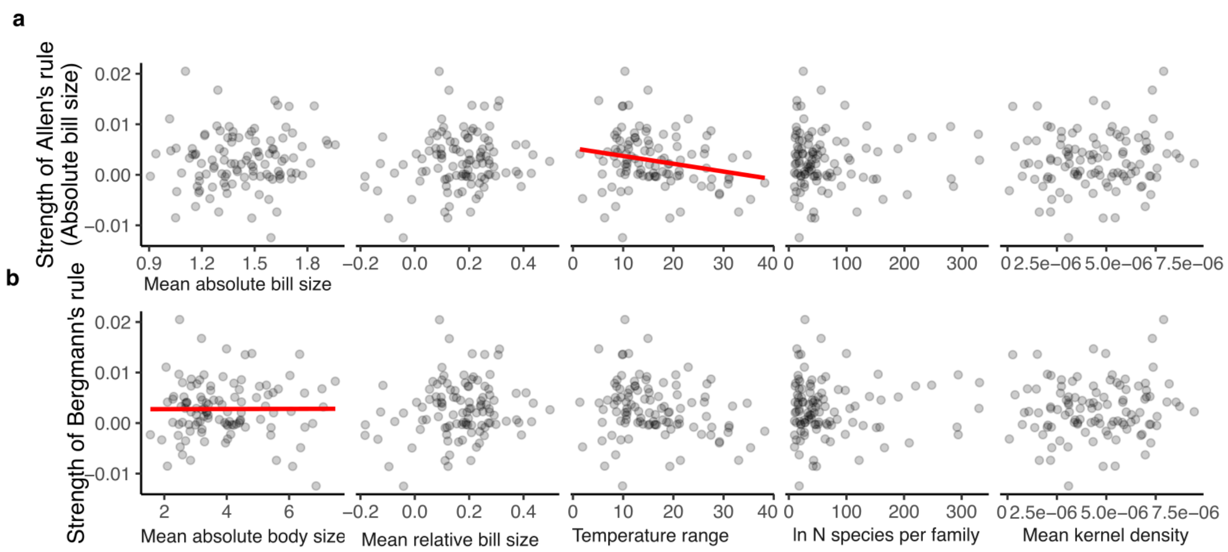

Supplementary Figure 10 | Multivariate regression of family-level traits against the strengths of conformity to Bergmann's and Allen's rules. Two separate phylogenetic regression models[9], each with N = 107, were used to explain variation in the family-level slopes originally estimated for our Allen's rule (**a**) and Bergmann's rule (**b**) analyses. Significant relationships are depicted in red. Specifically, temperature range had a significant effect on the slope of Allen's rule (effect size = -0.0013, SE = 0.0006, P = 0.022), and mean absolute body size had a significant effect on the slope of Bergmann's rule (effect size = 0.0206, SE = 0.0034, P = 0.0008).

Supplementary References

1. Jetz, W., Thomas, G. H., Joy, J. B., Hartmann, K. & Mooers, A. O. The global diversity of birds in space and time. *Nature* **491**, 444–448 (2012).

2. Hackett, S. J. *et al.* A phylogenomic study of birds reveals their evolutionary history. *Science* **320**, 1763–1768 (2008).

3. Agresti, A. *Categorical Data Analysis*. (John Wiley & Sons, 2002).

4. Tobias, J. A. *et al.* AVONET: morphological, ecological and geographical data for all birds. *Ecol Lett* **25**, 581–597 (2022).

5. Tattersall, G. J., Andrade, D. V. & Abe, A. S. Heat exchange from the toucan bill reveals a controllable vascular thermal radiator. *Science* **325**, 468–470 (2009).

6. Powers, D. R., Tobalske, B. W., Wilson, J. K., Arthur Woods, H. & Corder, K. R. Heat dissipation during hovering and forward flight in hummingbirds. *R Soc Open Sci* **2**, (2015).

7. Baldwin, J. W. Garcia Porta, J. & Botero C. A. Data and Code: Complementarity in Allen's and Bergmann's rules among birds. *Zenodo Dataset*, Version **1.4**. https://zenodo.org/record/8092265 (2023).

8. Ericson, P. G. P. *et al.* Diversification of Neoaves: Integration of molecular sequence data and fossils. *Biol Lett* **2**, 543–547 (2006).

9. Tung Ho, L. S. & Ané, C. A linear-time algorithm for gaussian and non-gaussian trait evolution models. *Syst Biol* **63**, 397–408 (2014).
