# Supplementary material for: Complementarity in Allen’s and Bergmann’s rules among birds
Source: Nat Commun. 2023 Jul 15;14:4240. doi: 10.1038/s41467-023-39954-9 (PMC10349823; doi:10.1038/s41467-023-39954-9)
Supplement: Supplementary file 3 — Reporting Summary [file 41467_2023_39954_MOESM3_ESM.pdf]

nature portfolio

Corresponding author(s): Justin W. Baldwin

Last updated by author(s): May 11, 2023

Reporting Summary

Nature Portfolio wishes to improve the reproducibility of the work that we publish. This form provides structure for consistency and transparency in reporting. For further information on Nature Portfolio policies, see our Editorial Policies and the Editorial Policy Checklist.

Statistics

For all statistical analyses, confirm that the following items are present in the figure legend, table legend, main text, or Methods section.

n/a	Confirmed	
☐	☒	The exact sample size (*n*) for each experimental group/condition, given as a discrete number and unit of measurement
☐	☒	A statement on whether measurements were taken from distinct samples or whether the same sample was measured repeatedly
☐	☒	The statistical test(s) used AND whether they are one- or two-sided<br>*Only common tests should be described solely by name; describe more complex techniques in the Methods section.*
☐	☒	A description of all covariates tested
☐	☒	A description of any assumptions or corrections, such as tests of normality and adjustment for multiple comparisons
☐	☒	A full description of the statistical parameters including central tendency (e.g. means) or other basic estimates (e.g. regression coefficient) AND variation (e.g. standard deviation) or associated estimates of uncertainty (e.g. confidence intervals)
☐	☒	For null hypothesis testing, the test statistic (e.g. *F*, *t*, *r*) with confidence intervals, effect sizes, degrees of freedom and *P* value noted<br>*Give P values as exact values whenever suitable.*
☐	☒	For Bayesian analysis, information on the choice of priors and Markov chain Monte Carlo settings
☐	☒	For hierarchical and complex designs, identification of the appropriate level for tests and full reporting of outcomes
☐	☒	Estimates of effect sizes (e.g. Cohen's *d*, Pearson's *r*), indicating how they were calculated

Our web collection on statistics for biologists contains articles on many of the points above.

Software and code

Policy information about availability of computer code

Data collection	All analyses were performed in R statistical software version 4.1.2. We used R packages MCMCglmm for main models and ggplot2 and ggtree for most visualization. For additional details, see code on Zenodo.
Data analysis	All code is provided on Zenodo: https://zenodo.org/record/7886396

For manuscripts utilizing custom algorithms or software that are central to the research but not yet described in published literature, software must be made available to editors and reviewers. We strongly encourage code deposition in a community repository (e.g. GitHub). See the Nature Portfolio guidelines for submitting code & software for further information.

Data

Policy information about availability of data

All manuscripts must include a data availability statement. This statement should provide the following information, where applicable:

- Accession codes, unique identifiers, or web links for publicly available datasets
- A description of any restrictions on data availability
- For clinical datasets or third party data, please ensure that the statement adheres to our policy

No new data were generated for this study. Avian morphology data is available from linear measurements29 and landmarks39. Temperature data are available from EcoClimate30. Distribution and ranges are available from BirdLife International31. Phylogenetic data are available from the Global Bird Phylogeny32. The analytic data are available on Zenodo52.

Research involving human participants, their data, or biological material

Policy information about studies with human participants or human data. See also policy information about sex, gender (identity/presentation), and sexual orientation and race, ethnicity and racism.

Reporting on sex and gender	N/A
Reporting on race, ethnicity, or other socially relevant groupings	N/A
Population characteristics	N/A
Recruitment	N/A
Ethics oversight	N/A

Note that full information on the approval of the study protocol must also be provided in the manuscript.

Field-specific reporting

Please select the one below that is the best fit for your research. If you are not sure, read the appropriate sections before making your selection.

☐ Life sciences ☐ Behavioural & social sciences ☒ Ecological, evolutionary & environmental sciences

For a reference copy of the document with all sections, see nature.com/documents/nr-reporting-summary-flat.pdf

Ecological, evolutionary & environmental sciences study design

All studies must disclose on these points even when the disclosure is negative.

Study description	We analyzed variation in bird morphology along global temperature gradients using Bayesian phylogenetic mixed models with family-level, phylogenetic, random slopes and intercepts.
Research sample	We sampled adult morphology from Avonet and a study of bill shape with landmark data, and sampled species-wide mean temperature from EcoClimate and BirdLife International. We obtained phylogenetic data from the Global Bird Phylogeny.
Sampling strategy	We restricted our analyses to terrestrial, non-migrant (resident) birds. We restricted our sampling to families with at least 10 species, and performed extensive robustness analyses.
Data collection	No new data were collected for this study, so we omit comments on data collection.
Timing and spatial scale	The phenotypic databases have been amassed over many decades of collaborative work and are, for the purposes of our study, quite comprehensive. Therefore, while we acknowledge that prolonged collaboration and data collection will yield some additional species' data, we think it is unlikely that they will change the major conclusions. We focused on terrestrial species, so in that sense we restricted the spatial scale, and this was done because some global climate datasets for use in ecology/evolution can have biased/inaccurate measurements for oceanic climates.
Data exclusions	We excluded marine/pelagic species (see above). We also excluded migratory species, as these experience different climates in different parts of their range as they move across climates and seasons.
Reproducibility	Code is reproducible and on Zenodo: https://zenodo.org/record/7886396
Randomization	N/A
Blinding	N/A

Did the study involve field work? ☐ Yes ☒ No

Reporting for specific materials, systems and methods

We require information from authors about some types of materials, experimental systems and methods used in many studies. Here, indicate whether each material, system or method listed is relevant to your study. If you are not sure if a list item applies to your research, read the appropriate section before selecting a response.

Materials & experimental systems

n/a	Involved in the study
☒	☐ Antibodies
☒	☐ Eukaryotic cell lines
☒	☐ Palaeontology and archaeology
☒	☐ Animals and other organisms
☒	☐ Clinical data
☒	☐ Dual use research of concern
☒	☐ Plants

Methods

n/a	Involved in the study
☒	☐ ChIP-seq
☒	☐ Flow cytometry
☒	☐ MRI-based neuroimaging
